# Massage: A Primer for Nurses

**Published:** 1891-12

**Authors:** 


MASSAGE: A PRIMER FOR NURSES. By SARAH E. POST, M. D., Lecturer before the Training Schools for Nurses, connected with Bellevue, Mt. Sinai, St. Luke's, and Charity Hospitals, New York. Second edition, with seven original photographs; 12mo, pp. 47. New York: The Nightingale Publishing Company. 1891.

In this little volume the subject of Massage is treated in a manner that none but a master of the art could do. The author wastes not a single word, but with a lucidity born of intelligent practice, she imparts her knowledge so plainly that it would seem as if any good and healthy individual may almost at once become expert. The only drawback would be ignorance of human anatomy. Though especially for nurses, the book ought to be in every house — if for no other reason — to prevent mischief by ignorance.

